# LPCAT2 inhibits colorectal cancer progression via the PRMT1/SLC7A11 axis

**DOI:** 10.1038/s41388-024-02996-4

**Published:** 2024-04-11

**Authors:** Nan Cao, Fangmei Zhang, Jiang Yin, Jianlei Zhang, Xiqing Bian, Guopei Zheng, Nan Li, Ying Lin, Liyun Luo

**Affiliations:** 1 Department of Clinical Oncology, Taihe Hospital, Hubei University of Medicine, Shiyan, 442000 PR China; 2 Key Laboratory of Cancer Therapy Resistance and Clinical Translational Study, Shiyan, 442000 PR China; 3 Affiliated Cancer Hospital and Institute of Guangzhou Medical University, Guangzhou Key Laboratory of “Translational Medicine on Malignant Tumor Treatment”, Guangzhou, 510095 PR China; 4 School of Pharmacy, Macau University of Science and Technology, Macao, 999078 China

**Keywords:** Colorectal cancer, Prognostic markers

## Abstract

Colorectal cancer (CRC) has a high degree of heterogeneity and identifying the genetic information of individual tumor cells could help enhance our understanding of tumor biology and uncover potential therapeutic targets for CRC. In this study, we identified LPCAT2+ tumor cell populations with less malignancy than LPCAT2- tumor cells in human and mouse CRC tissues using scRNA-seq. Combining in vitro and in vivo experiments, we found that LPCAT2 could inhibit the proliferation of CRC cells by inducing ferroptosis. Mechanistically, LPCAT2 arrested PRMT1 in cytoplasm of CRC cells via regulating acetylation of PRMT1 at the K145 site. In turn, PRMT1 enhanced SLC7A11 promoter activity. Thus, LPCAT2 attenuated the positive regulatory effect of PRMT1 on SLC7A11 promoter. Notably, SLC7A11 acts as a ferroptosis regulator. Furthermore, in LPCAT2 knockout mice (LPCAT2−/−) colon cancer model, we found that LPCAT2−/− mice exhibited more severe lesions, while PRMT1 or SLC7A11 inhibitors delayed the progression. Altogether, we elucidated that LPCAT2 suppresses SLC7A11 expression by inhibiting PRMT1 nuclear translocation, thereby inducing ferroptosis in CRC cells. Moreover, inhibitors of the PRMT1/SLC7A11 axis could delay tumor progression in CRC with low LPCAT2 expression, making it a potentially effective treatment for CRC.

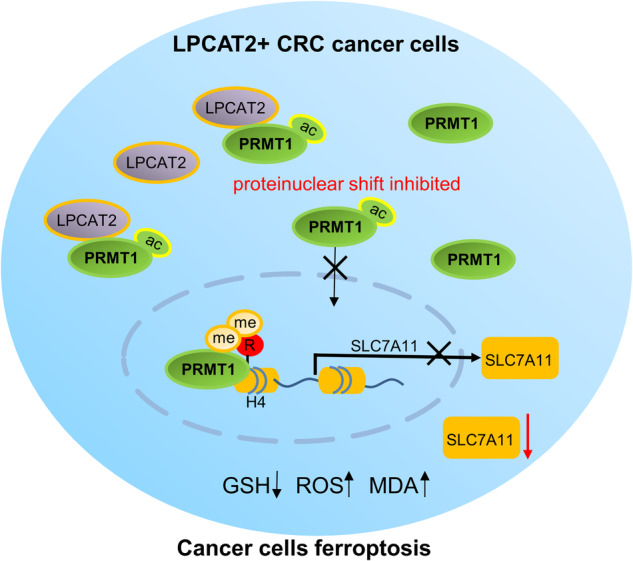

## Introduction

Colorectal cancer (CRC) is the third most common cancer worldwide, and its mortality rate ranks second in cancer-related deaths [1]. Classification schemes for CRC have been well-established based on the intrinsic features of tumor cells, such as histopathology, bulk gene expression, chromosomal instability, hypermethylation, and microsatellite instability. However, the mutational makeup of tumor cells alone does not enable us to subclassify tumor types for precise therapy or accurately predict patient survival [2]. The biological behavior of CRC is significantly impacted by the anatomical site of the tumor, thereby influencing its molecular biology, clinical presentations, and therapeutic interventions [3, 4]. Specifically, right-sided CRC (RCRC) is linked to the consensus molecular subtype 2 (CMS2) and exhibits a higher prevalence of DNA somatic copy number alterations (SCNA) as well as microsatellite stability/weak immune activation [5]. Consequently, this subtype demonstrates a relatively diminished response to immunotherapy. Left-sided CRC (LCRC) patients exhibit a more favorable prognosis compared to RCRC patients [6]. Consequently, elucidating the molecular disparities between these two subtypes holds immense importance in the advancement of therapeutic agents that target distinct segments of CRC.

Ferroptosis is a novel form of regulated cell death driven by excessive lipid peroxidation, and is considered to be a natural tumor suppression mechanism [7]. It has been proven to be associated with the development and therapeutic responses of liver cancer, lung cacner, CRC, breast cancer, pancreatic cancer, gastric cancer [8–10]. SLC7A11 (Solute Carrier Family 7 Member 11, also known as xCT) is a key component of cystine transporter and plays an increasingly essential role in regulating tumor ferroptosis [11]. PERK inhibition promotes ferroptosis by suppressing SLC7A11 in CRC [12]. SLC7A11 expression is regulated at multiple levels, such as transcription factors ATF4 and NRF2, which can directly transcriptionally regulate SLC7A11 expression [13, 14]. BAP1 and SWI/SNF regulate SLC7A11 expression through epigenetic regulation of histone modification and chromatin remodeling [15, 16].

In this study, a population of LPCAT2 + CRC cells in CRC was identified using single-cell RNA sequencing (scRNA-seq), demonstrating ferroptosis characteristics. LPCAT2, a member of the LPAAT family, is known to play a crucial role in regulating phospholipid metabolism and providing substrates for lipid peroxidation [17]. Previous studies have reported the involvement of LPCAT2 in ferroptosis in CRC cells [18], but the underlying molecular mechanisms remain unclear. LPCAT2 has been fully demonstrated to participate in the inflammatory process of non-alcoholic steatohepatitis [19, 20]. In relation to cancer, LPCAT2 has been reported to have either oncogenic or cancer-suppressive effects in various cancer types. In cervical cancer cells, LPCAT2 transactivated EGFR signaling and was associated with unfavorable prognosis [21]. Chemotherapy induced LPCAT2 expression resulting into lipid droplet accumulation and chemoresistance in CRC cells, but without effect on proliferation [22]. It seemed inconsistent with the role of lipid droplets in CRC stem-like cell self-renewal [23]. Other observations suggested a tumor-suppressive role for LPCAT2 in certain cancer types, such as papillary thyroid cancer. LPCAT2 was negatively associated with disease progression but positively associated with survival [24]. In light of these conflicting data, we sought to delineate the roles and molecular mechanisms of LPCAT2 in CRC tumorigenesis and progression using biological and clinical models.

Protein Arginine Methyltransferase 1 (PRMT1) is a member of the protein arginine methyltransferase (PRMT) family and primarily facilitates asymmetric dimethylation at H4 histone arginine 3 (H4R3me2a), which serves as a significant indicator for transcriptional activation [25]. Additionally, PRMT1 is involved in various biological processes including signal transduction, transcriptional regulation, and DNA repair [26]. It has been documented that PRMT1 accelerates adipose differentiation by upregulating expression of the transcription factor transcription factor peroxisome proliferator-activated receptor-γ (PPARγ) through the catalyzation of H4R3me2a [27]. Furthermore, PRMT1 has been found to facilitate arginine methylation of NONO, thereby enhancing its oncogenic functions in CRC [28]. Additionally, PRMT1 has been identified as a potential target for tumor therapy. Previous study has demonstrated that the downregulation of PRMT1 or inhibition of its enzymatic activity in the DSS-induced Apc^min/+^ CRC model resulted in a delay in CRC progression [29]. However, the interplay between LPCAT2 and PRMT1 in CRC has not been investigated. In this study, we hypothesize that PRMT1 functions as a transcriptional regulator. Our primary objective is to elucidate the mechanism by which LPCAT2 modulates the expression of SLC7A11 through PRMT1, ultimately leading to the induction of ferroptosis in CRC cells.

## Results

### scRNA-seq identifies LPCAT2-positive tumor cells in CRC

**Fig. 1:**
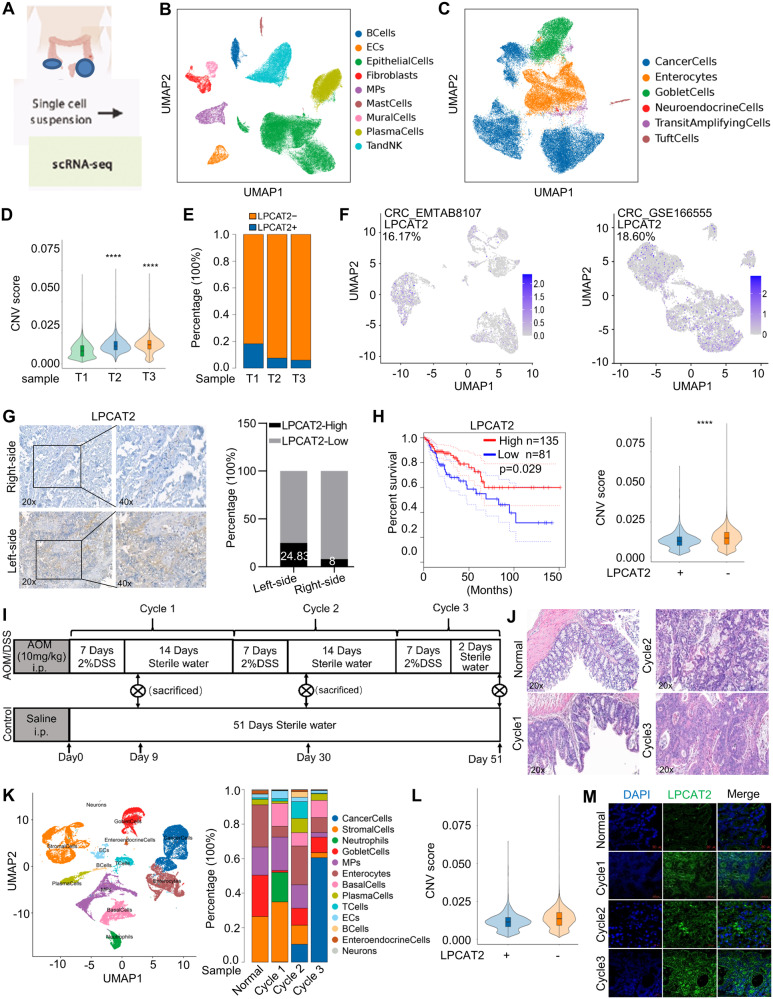
scRNA-seq identifies LPCAT2-positive tumor cell subsets. **A** Human colon cancer tissues were used for single-cell RNA sequencing. **B** UMAP plot. **C** Subdivision of epithelial cell subpopulations, UMAP plot of cells colored by cell type. **D** CNV score of LPCAT2+ and LPCAT2- cell populations. **E** Histogram showing the percentage of LPCAT2+ tumor cell subsets in different samples. **F** UMAP plot of LPCAT2 expression in the CRC_EMTAB8107 and CRC_GSE166555 datasets. **G** Immunohistochemistry for LPCAT2 in colon cancer tissues. **H** The relationship between LPCAT2 expression and prognosis in CRC was analyzed using the GEPIA database. **I** The experimental procedure for azoxymethane (AOM)/dextran sulfate sodium (DSS)-induced colorectal carcinogenesis in mice is outlined in the scheme. **J** HE staining clarified the intestinal histopathology at each time point. **K** The UMAP plot shows cell subpopulations and histograms display cell proportions in the AOM/DSS mouse model. **L** CNV values were measured for LPCAT2+ tumor cell subsets and LPCAT2- tumor cells in the mouse model. **M** Immunofluorescence was used to visualize LPCAT2 expression in the AOM/DSS tumor model, with a scale bar of 20 μm. ****P < 0.0001.

We obtained three CRC tissues immediately after therapeutic surgery. One sample was from the left-sided colon (marked as T1), and the other two were from the right-sided colon (marked as T2 and T3). Single-cell RNA sequencing (scRNA-seq) was performed to obtain transcriptomes of individual cells using the Singleron GEXSCOPE platform (Fig. 1A). Tumor tissues were classified into various cell types, including epithelial cells, mononuclear phagocytes, endothelial cells, plasma cells, fibroblasts, mast cells, mural cells, T and NK cells, and B cells, using Uniform Manifold Approximation and Projection (UMAP) for visualization (Fig. 1B; Supplementary Fig. 1A). Within the epithelial cell category, malignantly transformed tumor cells were identified by calculating the malignance score based on copy number vatiation (CNV) (Supplementary Fig. 1B). Cancer cells from the left-sided colon had a lower malignance score compared to cancer cells from the right-sided colon (Supplementary Fig. 1C). The dominant subsets within the epithelial cell population were found to be tumor cells (Fig. 1C; Supplementary Fig. 1D). Here, we mainly focused on tumor cells to explore its heterogeneity and molecular mechanisms responsible for CRC progression. We identified a population of LPCAT2-positive (LPCAT2 + ) tumor cells (Supplementary Fig. 1E and F) with a lower CNV score than LPCAT2-negative (LPCAT2-) population (Fig. 1D; Supplementary Fig. 1G). Surprisingly, we observed a significantly higher proportion of LPCAT2+ cells in the left-sided colon compared to the right-sided colon (Fig. 1E). Consistently, the LPCAT2+ cell subpopulation was also detected in scRNA-seq datasets of CRC_EMTAB8107 and CRC_GSE166555 respectively (Fig. 1F).

To further validate the LPCAT2 expression pattern in CRC, we detected LPCAT2 protein levels in 50 colon cancer tissues. The LPCAT2 expression in left-sided colon cancer tissues was higher than that in right-sided tissues (Fig. 1G). Additionally, TCGA data (http://gepia.cancer-pku.cn/) showed that LPCAT2 expression level was positively correlated with overall survival in CRC patients (Fig. 1H), and LPCAT2 expression was not associated with the mutation of TP53 (Supplementary Fig. 1H). Analysis based on CPTAC data also uncovered that there was no correlation between LPCAT2 level and Hippo or Myc status (Supplementary Fig. 1I).

To explore the dynamic changes in LPCAT2 expression during colon cancer progression, we established colitis-associated colon cancer (CAC) mouse model by challenging C57BL/6 mice with intraperitoneal injection of AOM and water-fed DSS (Fig. 1I). The colon tissues were collected at different time points during the course of CAC development, which presented as inflammation, low-grade dysplasia, high-grade dysplasia and canceration sequential pathological process (Fig. 1J; Supplementary Fig. 1J). Moreover, tissues from the corresponding time point were subjected to scRNA-seq analysis. Based on CNV analysis, cancer cells were identified from cycle2 and cycle3 time point tissues (Fig. 1K). Similarly, the LPCAT2+ subpopulation has a lower malignancy score than the LPCAT2- subpopulation in the CAC model (Fig. 1L). LPCAT2 expression in epithelial cells was dramatically increased in the inflammatory phase (Fig. 1M). These results suggested the potential protective effects of LPCAT2 in CRC development.

### LPCAT2 suppresses CRC proliferation

**Fig. 2:**
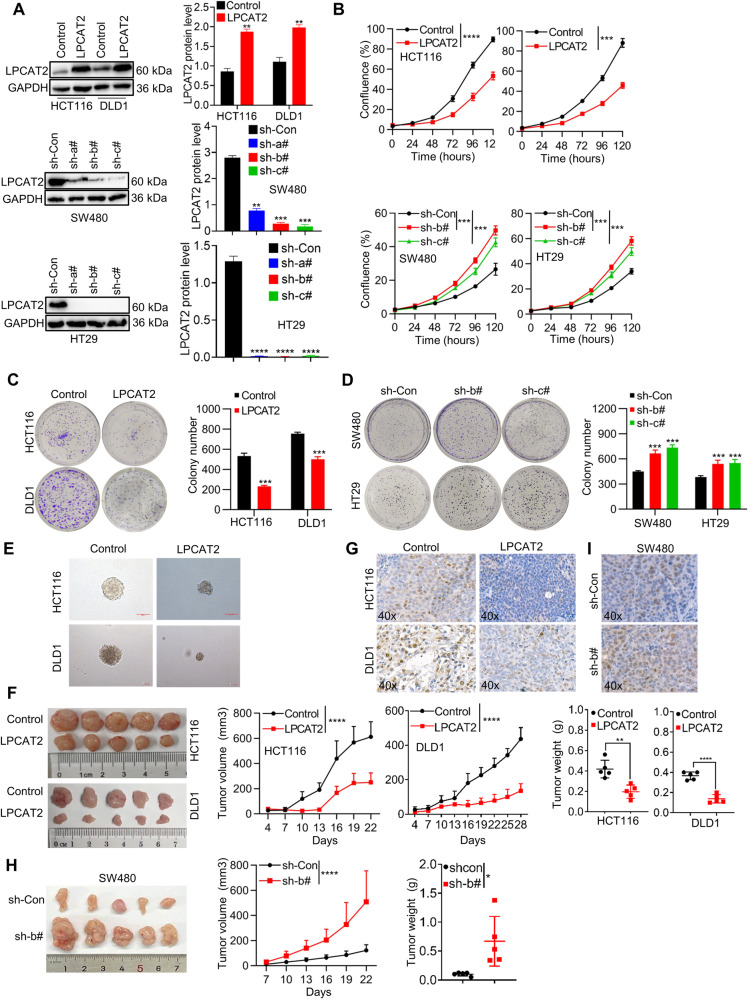
LPCAT2 influences the proliferation of CRC cells. **A** LPCAT2 expression in stable cell lines of CRC overexpressing or knocking down LPCAT2. **B**– **E** The function of interfering with LPCAT2 expression on CRC cells proliferation in vitro. Scale bar: 100 μm. **F**, **H** Subcutaneous tumor formation and tumor growth curves, tumor weight in nude mice overexpressing LPCAT2 (**F**) or LPCAT2 inhibition (**H**). **G**, **I** Expression of Ki67 in tumor tissue with differential expression of LPCAT2. *P < 0.05, ** *P* < 0.01, ***P < 0.001, ****P < 0.0001.

To evaluate the biological roles of LPCAT2 in CRC, we conducted an analysis of the endogenous expression of LPCAT2 in both human normal epithelial cell lines (FHC) and CRC cell lines (Supplementary Fig. 2A). We found that HCT116 and DLD1 showed lower LPCAT2 expression compared to other CRC cell lines, while SW480 and HT29 exhibited higher expression levels. Consequently, we opted to generate LPCAT2 overexpressing cell lines in HCT116 and DLD1 cells, while LPCAT2 low expression cell lines were established in SW480 and HT29 (Fig. 2A; Supplementary Fig. 2B). The cell proliferation was monitored using living cells workstation. As shown, LPCAT2 overexpression inhibited CRC cells proliferation (Fig. 2B). The colony-forming assay and soft agar assay also showed that LPCAT2 overexpression significantly inhibited the colony-formation ability of CRC cells (Fig. 2C and E). Conversely, LPCAT2 knockdown markedly promoted cells proliferation and colony-forming capacity (Fig. 2B and D). Moreover, we implemented subcutaneous injection of CRC cells with LPCAT2 overexpression or knockdown in nude mice. LPCAT2 overexpression repressed tumor growth compared to control (Fig. 2F), and also downregulated the expression level of proliferation marker Ki67 (Fig. 2G). On the contrary, LPCAT2 knockdown promoted CRC cells growth in vivo (Fig. 2H) and increased the Ki67 level in mice xenograft (Fig. 2I).

### LPCAT2 induces ferroptosis via inhibiting SLC7A11 expression

**Fig. 3:**
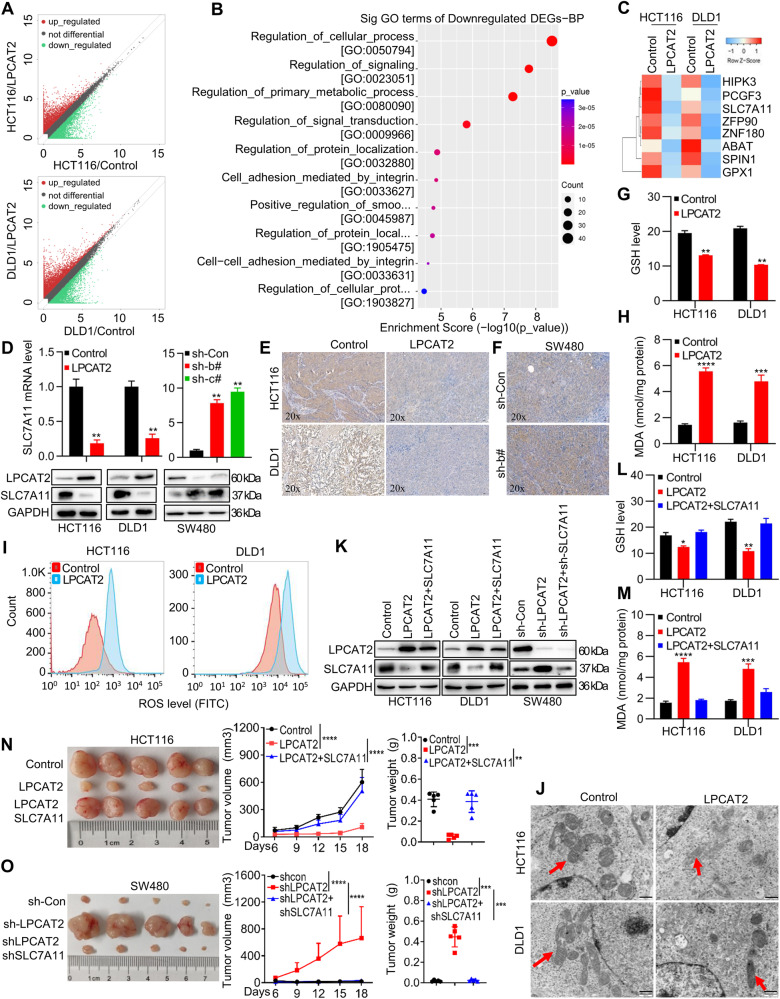
LPCAT2 induces ferroptosis in CRC cells by regulating SLC7A11. **A** Scatter plot showing differential gene expression in LPCAT2 overexpression CRC cells. **B** Bubble chart illustrating Gene Ontology (GO) enrichment analysis for commonly down-regulated differential genes. **C** Heatmap clustering of down-regulated differential genes. **D** Analysis of SLC7A11 mRNA and protein levels in CRC cell lines with interference of LPCAT2. **E**, **F** Immunohistochemistry (IHC) staining for SLC7A11 in subcutaneous tumor tissues with differential expression of LPCAT2. **G**, **H** Measurement of GSH (**G**) and MDA (**H**) content in LPCAT2 overexpression cells. **I** Intracellular ROS levels were detected using flow cytometry. **J** The morphology of mitochondria in cells overexpressing LPCAT2 was examined. Scale bars: 500 nm. **K** Stable CRC cell lines were constructed to interfere with LPCAT2 and SLC7A11, and WB assays were performed to verify the expression of target proteins. **L**, **M** The GSH (**L**) and MDA (**M**) content in the stable cell lines constructed in (**K**) was measured. **N**, **O** Functional rescue experiments of SLC7A11 were conducted in nude mice. *P < 0.05, **P < 0.01, ***P < 0.001, ****P < 0.0001.

To explore the molecular mechanisms underlying the tumor suppressive role of LPCAT2, we conducted RNA-sequencing analysis to identify differentially expressed genes (DEGs) in CRC cells overexpressing LPCAT2 (Fig. 3A). There were 33 genes commonly upregulated and 66 genes commonly downregulated (Supplementary Fig. 3A). To refine our gene screening approach, we performed an intersection analysis between the top 240 differentially downregulated genes in HCT116 and DLD1 cells that exhibited high LPCAT2 expression (Supplementary Fig. 4A). GO enrichment analysis showed that the downregulated DEGs were mainly associated with metabolic process (Fig. 3B; Supplementary Fig. 4B). Subsequently, the first 240 co-downregulated genes were intersected with DEGs involved in metabolic regulation, resulting in the identification of 8 DEGs (Supplementary Fig. 4C). A gene expression heatmap was generated to visualize the expression patterns (Fig. 3C). LPCAT2 has been reported to play a role in the regulating ferroptosis, although its underlying molecular mechanism remains unclear. Intriguingly, SLC7A11 expression was significantly decreased upon LPCAT2 overexpression (Fig. 3C). SLC7A11 is an essential component of cystine transporter and acts as a key suppressor of ferroptosis. This led us to propose that metabolic disturbance, probably ferroptosis induced by SLC7A11 might be regulated differently in LPCAT2 expressed CRC cells. We confirmed that LPCAT2 negatively regulated SLC7A11 expression at mRNA and protein levels in CRC cells (Fig. 3D; Supplementary Fig. 4D). However, there was no expression change of other ferroptosis related genes such as GPX4, ALOX15 or NCOA4 (Supplementary Fig. 3B). LPCAT2 repressing SLC7A11 expression was also validated in xenograft tissues of nude mice (Fig. 3E, F). Since SLC7A11 regulates the uptake of extracellular cystine, a major precursor for GSH biosynthesis, we found that LPCAT2 overexpression significantly decreased the levels of GSH (Fig. 3G). Ferroptosis is typically characterized by intracellular ROS accumulation and lipid peroxidation, while malondialdehyde (MDA) is a well-established biomarker of lipid peroxidation. LPCAT2 overexpression in basal conditions or in combination with ferroptosis inducer erastin notably elevated lipid peroxide MDA (Fig. 3H; Supplementary Fig. 5A) and the ROS level (Fig. 3I). Whereas, LPCAT2 knockdown increased the levels of GSH (Supplementary Fig. 3C) but decreased MDA level (Supplementary Fig. 3D; Supplementary Fig. 5A). Transmission electron microscopy (TEM) analysis further revealed that LPCAT2 overexpression promoted mitochondria shrinkage with elevated membrane density, a typical morphological feature of ferroptosis (Fig. 3J). To verify that LPCAT2 suppressed CRC cells proliferation via inhibiting SLC7A11, we ectopically re-expressed in CRC cells with LPCAT2 overexpression and knocked it down in CRC cells with low LPCAT2 expression (Fig. 3K; Supplementary Fig. 4E). The re-expression of SLC7A11 rescued the GSH (Fig. 3L) and MDA levels (Fig. 3M) induced by LPCAT2 overexpression. Conversely, the knockdown of SLC7A11 reversed the increase in GSH levels mediated by LPCAT2 knockdown (Supplementary Fig. 3E) and the decrease in MDA levels (Supplementary Fig. 3F).

Biologically, SLC7A11 restoration effectively blocked the proliferation inhibition mediated by LPCAT2 overexpression in vitro and in vivo (Supplementary Fig. 3G and H; Fig. 3N). Conversely, SLC7A11 knockdown in LPCAT2-low expressing CRC cells undermined the effect of LPCAT2 knockdown on proliferation in vitro and in vivo (Supplementary Fig. 3I; Fig. 3O). These results suggested that LPCAT2 exerted its tumor suppressor role via promoting ferroptosis by inhibiting SLC7A11 expression.

### LPCAT2 prevents PRMT1 nuclear translocation

**Fig. 4:**
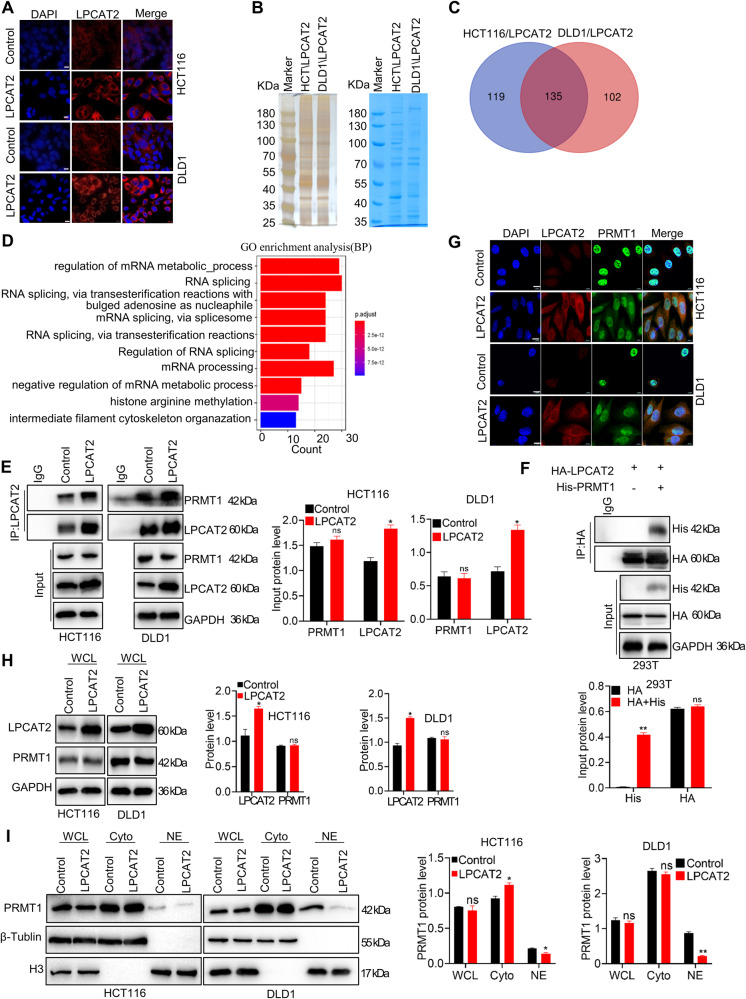
LPCAT2 binds to the PRMT1 protein. **A** LPCAT2 protein localization was observed by immunofluorescence. Scale bar: 10 um. **B** SDS-page gel for silver staining (left) and Coomassie brilliant blue staining (right). **C** Venn diagram of binding proteins taken from HCT116-LPCAT2 group and DLD1-LPCAT2 group. **D** GO enrichment analysis of the common binding proteins in HCT116-LPCAT2 group and DLD1-LPCAT2 group. **E** Immunoprecipitation (IP) of LPCAT2 with LPCAT2 antibody was performed to verify the interaction between LPCAT2 and PRMT1. **F** Immunoprecipitation (IP) for LPCAT2 protein tagged with HA tag using anti-HA antibody, followed by western blotting. **G** The cellular distribution of PRMT1 in LPCAT2 overexpression cells using immunofluorescence. Scale bar: 10 um. **H**, **I** The nucleoplasmic separation experiments denoted cellular allocation of PRMT1 in CRC cells overexpressing LPCAT2. *P < 0.05, ns indicates no statistical significance.

In order to gain insight into the mechanism underlying the action of LPCAT2 in CRC cells, we initially established that LPCAT2 predominantly localizes in the cytoplasm (Fig. 4A). Considering that transcriptional regulation typically takes place in the nucleus, it is reasonable to infer that LPCAT2 may modulate SLC7A11 expression through an intermediary protein. Subsequently, we employed immunopurification and mass spectrometry analysis to identify proteins associated with LPCAT2 in CRC cell lines overexpressing LPCAT2 (Fig. 4B), and 135 common binding proteins were found (Fig. 4C). GO enrichment analysis showed that LPCAT2-associated proteins were involved in histone arginine methylation pathway (Fig. 4D). Among these proteins, we focused on PRMT1, which plays a role in transcriptional control during normal or disease development. We hypothesized that LPCAT2 may regulate SLC7A11 expression by interacting with PRMT1. To confirm this interaction, reciprocal co-immunoprecipitation was performed, which confirmed the binding between LPCAT2 and PRMT1 (Fig. 4E, F). Interestingly, LPCAT2 did not have an impact on the protein expression of PRMT1 (Fig. 4E, F). Additionally, immunofluorescence staining revealed that LPCAT2 co-located with PRMT1 in the cytoplasm and LPCAT2 seemed to partially arrest PRMT1 in cytoplasm (Fig. 4G). Moreover, PRMT1 protein level in nucleus declined in the LPCAT2 overexpression groups using nucleoplasmic separation experiments (Fig. 4H, I).

### PRMT1 K145 acetylation modification is responsible for LPCAT2-mediated inhibition of nuclear translocation

**Fig. 5:**
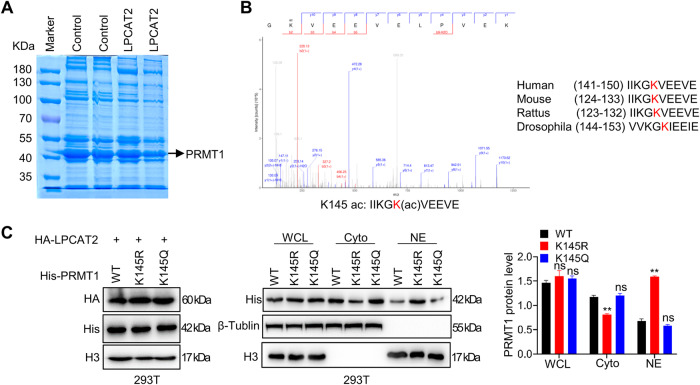
LPCAT2 participates in the acetylation modification of PRMT1 K145 site. **A** PRMT1 protein was enriched by coIP in HCT116 overexpression LPCAT2 followed by SDS-page gel Coomassie brilliant blue staining. **B** Mass spectrometric identification of PRMT1 protein modification sites. **C** HA-LPCAT2, His-PRMT1 wild-type or mutant plasmids were co-transfected into 293 T cells. The distribution of PRMT1 protein in cells was detected by nucleoplasmic separation experiments, and the grayscale of protein bands was analyzed by ImageJ. *P < 0.05, **P < 0.01.

Given that LPCAT2 did not alter PRMT1 protein levels, we speculated whether PRMT1 underwent post-translational modification. PRMT1 protein was immunoprecipitated from HCT116 cells with LPCAT2 overexpression, and analyze the gel where PRMT1 protein located by LC-MS/MS (Fig. 5A). Mass spectrometry showed that PRMT1 K145 site was acetylated (Fig. 5B). We guessed that LPCAT2 may regulate subcellular localization of PRMT1 through acetylating PRMT1 K145 site. To investigate the interaction between LPCAT2 and PRMT1, we generated PRMT1 K145 site mutants (PRMT1 K145R, PRMT1 K145Q) and co-transfected LPCAT2 and PRMT1 vector in HEK293T cells. Upon LPCAT2 overexpression, the distribution of PRMT1-K145R proteins in the cytoplasm was reduced compared to WT proteins, with a corresponding increase in nuclear distribution (Fig. 5C). These findings suggested that LPCAT2 could induce acetylation modification at PRMT1 K145 site, thereby blocking PRMT1 protein in the cytoplasm.

### LPCAT2 inhibits SLC7A11 expression via PRMT1 protein modulation

**Fig. 6:**
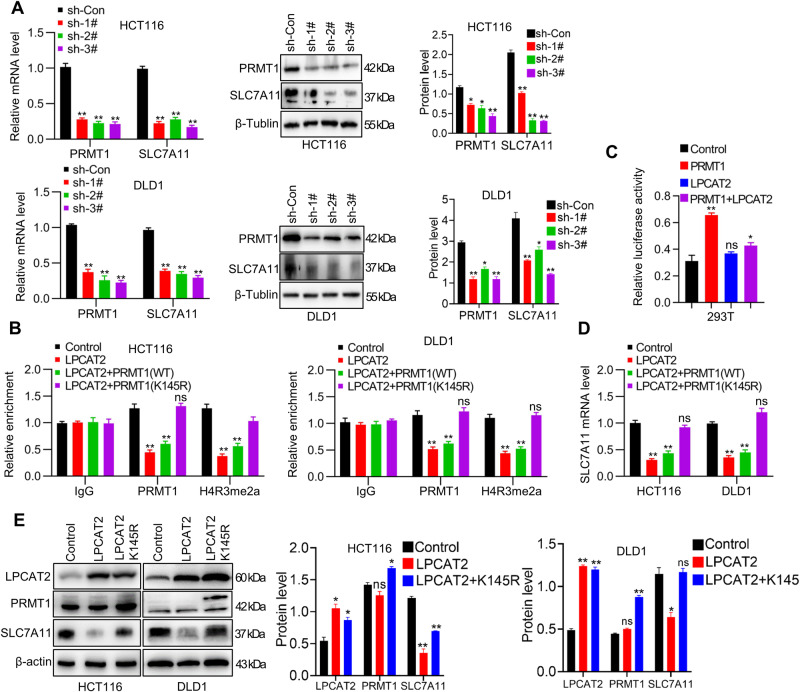
LPCAT2 regulates SLC7A11 expression through acetylation of PRMT1 at the K145 site. **A** Expression of PRMT1 and SLC7A11 in CRC cells interfered with PRMT1. **B** Fold change of PRMT1 and H4R3me2a enrichment at the SLC7A11 promoter in CRC cells detected by ChIP-PCR. **C** Dual luciferase reporter assay. **D**, **E** SLC7A11 expression in LPCAT2 overexpression cells transfected with PRMT1 wild-type or K145 mutant plasmid was detected using qRT-PCR (**D**) or WB (**E**). *P < 0.05, **P < 0.01.

Considering that LPCAT2 not only down-regulated SLC7A11 expression but also affected the nucleoplasmic distribution of PRMT1 proteins, we further investigated whether LPCAT2 inhibited SLC7A11 via modulating PRMT1 protein. Firstly, PRMT1 knockdown decreased SLC7A11 expression in CRC cells (Fig. 6A), but did not affect the expression of LPCAT2 (Supplementary Fig. 5C). Considering that PRMT1 is an arginine methyltransferase that regulats asymmetric dimethylation of histone H4R3 (H4R3me2a). Thus, we detected the enrichment of PRMT1 and H4R3me2a at SLC7A11 promoter. Expectedly, LPCAT2 overexpression decreased PRMT1 and H4R3me2a enrichment at the SLC7A11 promoter, while the PRMT1 K145 mutant obviously rescued H4R3me2a levels at the SLC7A11 promoter (Fig. 6B). Furthermore, the potentiation of PRMT1 on SLC7A11 promoter was counteracted by LPCAT2, which did not directly regulate SLC7A11 promoter activity (Fig. 6C). We also found that the PRMT1 K145 mutant restored the expression of SLC7A11, which had been reduced by LPCAT2 overexpression (Fig. 6D, E).

### LPCAT2/PRMT1/SLC7A11 axis is involved in CRC tumorigenesis and clinical prognosis

**Fig. 7:**
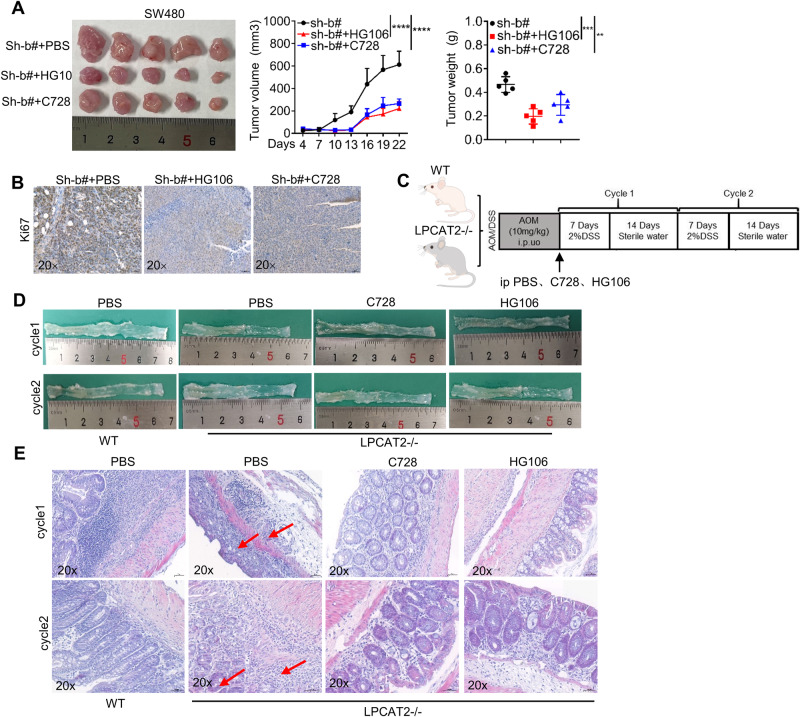
PRMT1/SLC7A11 axis inhibitor delays tumor progression in LPCAT2 low-expressing CRC cells. **A** Therapeutic effects of C728 and HG106 in mice bearing low-expressing LPCAT2 CRC cells. **B** Ki67 expression in tumor tissues. **C** Schematic diagrams of colitis-associated colon cancer modeling in WT and LPCAT2−/− mice. **D** Histomorphology of the colon in mice receiving different treatments at different time periods. **E** HE staining of colon tissues. **P < 0.01, ***P < 0.001, ****P < 0.0001.

Given that CRC cells with low LPCAT2 expression showed more malignancy than those with high LPCAT2 expression, we sought to examine whether inhibition of PRMT1 and SLC7A11 would have a favorable therapeutic effect on LPCAT2 low expression CRC cells. We initially examined the effects of PRMT1 inhibitor, C-7280948 (referred to as C728) and SLC7A11 inhibitor HG106 in vivo. We established LPCAT2 low expression SW480 cell line-derived xenograft (CDX) tumor models. Tumor-bearing mice were treated with intraperitoneal injections of C728 or HG106 for 22 days. It was shown that C728 or HG106 had a moderate therapeutic effect (Fig. 7A), and Ki67 expression was also decreased in tissues treated with C728 or HG106 (Fig. 7B). To further verify therapeutic effect of C728 or HG106 in CRC with low LPCAT2 expression, a colitis-associated colon cancer (CAC) mouse model was constructed in LPCAT2−/− mice (Fig. 7C). Mice colon length was longer with C728 or HG106 treatment (Fig. 7D). Uniformly, LPCAT2−/− mice treated with PBS exhibited more severe lesions than WT group, with colon epithelial atrophy and obvious epithelial hyperplasia, with lesions breaking through the basement membrane (shown by red arrows on Fig. 7E), while the C728 and HG106 treatment groups showed milder lesions (Fig. 7E). It suggested that a therapeutic role for C728 and HG106 in CRC cells with low LPCAT2 expression.

## Discussion

CRC is highly heterogeneous, which is a major factor in the difference in tumor prognosis [30, 31]. scRNA-seq has been employed to investigate tumor heterogeneity [32, 33]. We identified a subpopulation of LPCAT2+ tumor cells via scRNA-seq analysis, which had a lower CNV score than LPCAT2- tumor cells. In addition, the GEPIA database showed that CRC patients with high LPCAT2 expression had a better prognosis. However, the role of LPCAT2 in cancer progression remains controversial. Numerous studies increasingly suggested that LPCAT2 acted as either an oncogene or a tumor suppressor in a cell-type and context-specific manner [21, 22, 24]. Here, we clarified that LPCAT2, upregulated in CRC cells, served as a protector to inhibit CRC cells proliferation in vitro and vivo, while inhibiting LPCAT2 expression had the opposite function.

LPCAT2 has been reported to have acetyltransferase activity [34]. Abate et al. found that the overall level of pan-lysine acetylation in RAW264.7 cells was significantly increased with LPS treatment, but LPCAT2 knockdown reduced it. Furthermore, they also found that LPCAT2 shared at least 65% similarity with lysine acetyltransferases KAT2A and KAT2B, suggesting that LPCAT2, KAT2A and KAT2B may be derived from the same superfamily [35]. Interestingly, we found that LPCAT2 could bind to PRMT1 and induced PRMT1 acetylation at K145 to arrest PRMT1 in cytoplasm. H4R3 is the first identified target of PRMT1, while asymmetric dimethylation of H4R3 (H4R3me2a) is associated with transcriptional activation [36, 37]. Here, we reported that PRMT1 mediated H4R3me2a modification on SLC7A11 promoter and enhanced its activity, which was attenuated in LPCAT2-overexpressed CRC cells.

SLC7A11 acts as a link between cancer metabolic disorders and ferroptosis [38]. As a critical regulator of ferroptosis, SLC7A11 could promote cancer progression partly through suppressing ferroptosis. For example, RBMS1 regulated lung cancer ferroptosis through translational control of SLC7A11 [39]. The p53 mutant (3KR) remained its tumor-suppressive function partially through inhibiting SLC7A11 and thus inducing ferroptosis [40]. Ferroptosis is typically characterized by mitochondria shrinking, increased membrane density, and elevated levels of reactive oxygen species (ROS), Malondialdehyde (MDA), and decreased intracellular glutathione (GSH) content [41]. Transmission electron microscopy showed that mitochondrial cristae were reduced or even disappeared in LPCAT2 high-expressing CRC cells. Furthermore, LPCAT2 overexpressing CRC cells had higher levels of ROS and MDA, and significantly lower GSH content, whereas LPCAT2 low-expressing CRC cells displayed the opposite trend. Recently, Carsten Culmsee et al. also reported the phenomenon of LPCAT2 driven ferroptosis [18]. These results indicated that redox homeostasis was disrupted, leading to lipid peroxide accumulation in LPCAT2 overexpressing CRC cells. We also discovered that PRMT1 or SLC7A11 inhibitors exerted profound anti-neoplastic activity against CRC cells overexpressing LPCAT2 in vivo.

To summarize, our study uncovered the heterogeneity of CRC cells with differential expression of LPCAT2. LPCAT2 + CRC cells showed less malignant than LPCAT2- CRC cells. LPCAT2 functioned as a novel ferroptosis inducer via PRMT1/SLC7A11 axis, inhibiting CRC cells proliferation. The LPCAT2/PRMT1/SLC7A11 axis is a promising target for CRC therapy.

## Methods

### CRC specimen collection

CRC tissues were collected from patients with pathological diagnosis of colorectal adenocarcinoma at the Affiliated Cancer Hospital of Guangzhou Medical University. The study was approved by the Clinical Research Ethics Committee of the Affiliated Cancer Hospital and Institute of Guangzhou Medical University. All patients provided informed consent to provide the samples, and none of them received chemotherapy, radiation, or immunotherapy prior to tumor resection.

### Single-cell RNA sequencing (scRNA-seq)

The fresh colon tissues were stored in the sCelLiveTM Tissue Preservation Solution (Singleron Biotechnologies, China) at 4 °C. The scRNA-seq libraries were constructed using the GEXSCOPE® Single-Cell RNA Library Kit and Singleron Matrix® Automated single-cell processing system, following the Singleron GEXSCOPE® operation instructions. Individual libraries were diluted to 4 nM and sequenced with 150 bp paired-end reads on Illumina Novaseq6000.

### Cells culture and transfection

Cells used in the study were purchased from American Type Culture Collection and cultured in DMEM medium (Gibco, USA) containing 10% fetal bovine serum (Gibco, USA) and 1% penicillin/streptomycin (Genom, China). Cell lines were reauthenticated with short tandem repeat analysis every 6 months after thawing in our experiments. All cells were incubated in a humidified incubator with 5% CO_2_ at 37 °C. Expression plasmids for LPCAT2 and LPCAT2 shRNA, PRMT1 shRNA were purchased from GeneCopoeia (Guangzhou, China). Lipo8000 reagent (Beyotime Biotechnology, China) was used for transient transfection. Puromycin (2.5 μg/ml) was utilized to select LPCAT2 overexpression or knockdown CRC cell lines.

### Cell proliferation assays

CRC cells were seeded into 96-well plates (2000 cells per well), and cell proliferation was monitored in real-time using a live cell workstation. For the soft agar assay, 1000 CRC cells were seeded in 6-well plates containing 0.35% top low-melt agarose and 0.7% bottom low-melt agarose. In the plate cloning experiment, 800 CRC cells were counted and inoculated into a 6-well plate.

### Dual-luciferase reporter assay

The gene encoding firefly luciferase was controlled by SLC7A11 promoter, while the gene encoding renilla promoter was fused to a constitutive TK promoter (Genechem, China). SLC7A11 promoter reporter plasmid system was co-transfected with PRMT1 plasmid (PLVX-IRES-NEO, Genecefe Biotechnology, China) or LPCAT2 plasmid (EX-Z1247-Lv120, GeneCopoeia, China) using lipo8000. After 24 hours, the promoter activity was determined using the dual luciferase assay kit (Vazyme, China). The relative activity was expressed as the ratio of firefly to renilla luciferase activity.

### RNA extraction and quantitative Real-time PCR (RT-qPCR)

Total RNA was extracted using trizol (Invitrogen, USA). cDNA was synthesized with first strand cDNA synthesis kit (Thermo Scientific, USA). Using 2×SYBR Green qPCR Master Mix (Selleckchem, USA) for real-time PCR and performed on CFX96 Real-Time PCR System (Bio-Rad, USA). Primers were synthesized by BGI Genomics (Shenzhen, China), primer pairs used are shown in supplementary methods.

### RNA sequencing

Total RNA was extracted and subjected to agarose electrophoresis and Nanodrop for quality control. NEB Next^®^ Poly(A) mRNA Magnetic Isolation Module (NEB, USA) was used for mRNA enrichment. Using KAPA Stranded RNA-Seq Library Prep Kit (Illumina, USA) to construct the library. Agilent 2100 inspect quality of the library. Using Illumina Novaseq 6000 sequencer for sequencing.

### Protein extraction and Western Blotting (WB)

Cell lysates were prepared on ice using RIPA lysis buffer (Boyotime, China) supplemented with 100x PMSF (Thermo Scientific, USA). WB was performed with the specific antibody, antibodies are shown in supplementary methods. The grayscale analysis of protein bands was conducted using the ImageJ software. The relative protein level = gray value of target protein bands / the corresponding internal reference gray value.

### Nuclear and cytoplasmic protein extraction

Nuclear and cytoplasmic proteins were extracted separately using the Nuclear and Cytoplasmic Protein Extraction Kit (Beyotime Biotechnology, China).

### HE and immunohistochemistry (IHC)

The CRC specimens and mouse samples were embedded in paraffin after dehydration. Hematoxylin and eosin (HE) staining was performed after the tissue sections were dewaxed and rehydrated. Antigen retrieval was performed using citrate buffer, followed by immunohistochemistry using the IHC UltraSensitiveTM SP kit (MXB Biotechnologies, China) and incubating specific primary antibodies at 4 °C overnight. Next, the tissues were stained with DAB reagents (MXB Biotechnologies, China) and scanned using a Pannoramic scanner (Pannoramic SCAN, Hungary).

### Co-IP and LC-MS/MS

Total protein was extracted using IP lysis buffer and incubated with specific antibodies overnight at 4 °C. The protein mixture was mixed with Protein A/G magnetic beads (BEAVER, China) incubating at 4 °C for 3 hours. The beads were then collected and washed three times, and finally binding protein was eluted with loading buffer. The precipitated protein mixtures were used for IP or LC-MS/MS analysis.

### Intracellular ROS, malondialdehyde (MDA), and glutathione (GSH) assay

ROS were detected using reactive oxygen species assay kit (Beyotime, China). The content of MDA was monitored using LPO MDA assay kit (Beyotime, China). The GSH detection kit (Solarbio Science, China) was used to detect GSH in CRC cells.

### ChIP-PCR assay

CRC cells were cross-linked with 1% formaldehyde for 10 minutes and then mixed with 0.125 M glycine to terminate the process. Follow-up procedures were carried out using the SimpleChIP® Plus Enzymatic Chromatin IP Kit (CST, USA) as per the reagent specifications. DNA was extracted and purified for subsequent PCR.

### Transmission electron microscopy

The cells were fixed in 2.5% glutaraldehyde (pH 7.4) for 2 hours, then rinsed three times with 0.1 M phosphate buffer (pH 7.2) and fixed with 1% osmic acid for 2 hours at 4 °C. After washing three times, the cells were dehydrated using a gradient of ethanol. Subsequently, the samples were embedded in Epon-Araldite resin for penetration and placed in a mold for polymerization. The embedded blocks were then ultrathin sectioned and counterstained with 3% uranyl acetate and 2.7% lead citrate. Finally, cell mitochondria were photographed using a transmission electron microscope.

### Animal studies

LPCAT2 heterozygous mice (C57BL/6 strain, female) were purchased from Gempharmatech (JiangSu, China), and LPCAT2 whole-body knockout mice (LPCAT2−/−) were constructed. WT and LPCAT2−/− mice were treated with AOM/DSS to establish CAC model. Briefly, 7-week-old female LPCAT2−/− mice and WT mice were intraperitoneally injected with AOM (10 mg/kg). After 7 days, the mice were given 2% DSS water for a week followed by sterile water for 14 days, repeating the process twice. LPCAT2−/− mice were treated with PRMT1 inhibitor C7280948 (15 mg/kg) or the SLC7A11 inhibitor HG106 (2 mg/kg) once daily at different periods of CAC model.

4-week-old female BALB/c nude mice were purchased from Gempharmatech and acclimatized for 1 week. 4 × 10^6^ CRC cells resuspended in 100 ul PBS were subcutaneously injected into the left axilla of nude mice, with five mice in each group. The tumor size was measured every 3 days, and tumor volume (V) was calculated as V = (length × width^2^)/2. The purchased mice were randomly assigned to different groups. The animal experiments were performed in an unblinded manner. All animal experiments conducted in this study were approved by the Institutional Animal Care and Use Committee of Guangzhou Medical University.

### Statistics analysis

All experiments were repeated three times independently, and GraphPad Prism 7.0 was used for data analysis. Power analysis was used to determine the sample sizes for the relevant experiments. Students’ t-tests were employed when the variance between two groups was similar, while Welch’s t-tests were utilized if the variance was not the same. The subcutaneous tumor growth curves were compared using two-way ANOVA. A p value less than 0.05 was considered statistically significant.

### Supplementary information

supplementary materials

## Data Availability

The datasets analysed during the study are available from the corresponding author.
