# Supplementary material for: LPCAT2 inhibits colorectal cancer progression via the PRMT1/SLC7A11 axis
Source: Oncogene. 2024 Apr 11;43(22):1714–25. doi: 10.1038/s41388-024-02996-4 (PMC11136653; doi:10.1038/s41388-024-02996-4)

**Supplementary methods**

**Single-Cell Preparation**

The fresh colon tissues were stored in the sCelLive^TM^ Tissue Preservation Solution (Singleron Biotechnologies, China) at 4°C. The specimens were washed three times with HBSS, minced into small pieces, and then digested with 3 mL sCelLive^TM^ Tissue Dissociation Solution (Singleron Biotechnologies, China) at 37 °C for 15 min using Singleron PythoN™ Tissue Dissociation System. The cell suspension was collected and filtered through a 40-micron sterile strainer. Subsequently, a 2-fold volume of GEXSCOPE^®^ erythrocytic lysis buffer (Singleron Biotechnologies, China) was added, and incubated at room temperature for 8 mins to remove erythrocytes. The mixture was then centrifuged at 300g, 4℃ for 5 mins to remove supernatant and gently suspended in PBS.

**Single-Cell RNA Sequencing (scRNA-seq)**

Single-cell suspensions were prepared with PBS (HyClone, Shanghai, China) at a cell concentration of 1 × 10^5^ cells/mL and loaded onto microfluidic chips. The scRNA-seq libraries were constructed using the GEXSCOPE^®^ Single-Cell RNA Library Kit and Singleron Matrix^®^ Automated single-cell processing system according to Singleron GEXSCOPE^®^ operation instructions. Individual libraries were diluted to 4 nM and sequenced with 150 bp paired end reads on Illumina novaseq6000.

**Quality control and analysis of Single-Cell RNA Sequencing data**

Raw data from sequencing were used to generate gene expression matrices using Singleron's internal analysis process. In brief, reads1 without poly T were filtered out, valid cell barcode and UMI were extracted; the reads2 filtered out of adapters and poly A tails (fastp V1) , then aligned and quantified against reference genomes in the ensembl database (STAR 2.5.3a and featureCounts 1.6.2). Reads, UMI and genes with the same cell barcode were grouped, and UMIs of each gene in each cell was calculated for subsequent analysis. Cell type identification and cluster analysis was performed on the RNA sequencing data using the Seurat program (http://satijalab.org/seurat/,R package,v.3.0.1). The expression matrix was imported into R using the read.table function, and cell clustering analysis was performed using the FindCluster function (parameter resolution 0.6). ClusterProfiler software was used for GO enrichment analysis.

**Primer pairs**

GAPDH F-5’ACAACTTTGGTATCGTGGAAGG3’, R-5’GCCATCACGCCACAGTTTC3’; LPCAT2 F-5’AGAAGCTCTGGGAATACCAGT3’, R-5’ATCCAAATGCTTACGAACACCA3’;SLC7A11 F-5’TCTCCAAAGGAGGTTACCTGC3’, R-5’AGACTCCCCTCAGTAAAGTGAC3’; PRMT1 F-5’CTTTGACTCCTACGCACACTT3’, R-5’GTGCCGGTTATGAAACATGGA3’.

ChIP-PCR for SLC7A11 primer pairs: F-5’CAGGACCTACCTACCTGTGG3’, R-5’TGCCAAACAAAAGCAAGCTGA3’.

**Antibodies**

LPCAT2 (1:1000, Abcam, ab224244), SLC7A11 (1:1000, Abcam, ab307601), PRMT1 (1:1000, Abcam, ab190892), GAPDH (1:8000, Proteintech, 60004-1-Ig), β-actin (1:1000, Proteintech, 20536-1-AP), β-tublin (1:10000, Abcam, ab179511), H3 (1:3000, Proteintech, 17168-1-AP).

**Supplementary Figure 1. scRNA-seq identifies LPCAT2-positive tumor cell subsets.**

**(A)** Percentage of cell subpopulations in human CRC tissues. **(B)** Heatmap showing CNV values of malignant epithelial cell populations in the right and left side of human CRC tissues. **(C)** CNV score for each sample (T1 indicates left-sided colon cancer tissue, T2 and T3 indicate right-sided colon cancer tissues). **(D)** Percentage of each subpopulation in the epithelial cells. **(E)** Pie chart of LPCAT2+ and LPCAT2- tumor cell subpopulations. **(F)** UMAP plot of LPCAT2 expression in malignant epithelial cell populations in human CRC tissues. **(G)** Heatmap showing CNV of LPCAT2+ and LPCAT2- tumor cells in AOM/DSS model. **(H** and **I)** Relationship between LPCAT2 expression and TP53 **(H)**, HIPPO and MYC mutation **(I)** in CRC tissues. **(J)** The colon tissues of AOM/DSS mouse model. **** P< 0.0001.

**Supplementary Figure 2. LPCAT2 influences the proliferation of CRC cells.**

**(A)** LPCAT2 expression in the normal intestinal epithelial cell line FHC and CRC cell lines. **(B)** mRNA expression of LPCAT2 in stable cell lines of CRC interfering with LPCAT2. ** P < 0.01, *** P < 0.001, **** P < 0.0001.

**Supplementary Figure 3. LPCAT2 induces ferroptosis in CRC cells through regulating SLC7A11.**

**(A)** The common differentially expressed genes in LPCAT2 high expression cells. **(B)** mRNA expression of GPX4, ALOX15 or NCOA4 in LPCAT2 differentially expressing cells. **(C-F)** GSH and MDA content in CRC cells. **(G-I)** Soft agar assay **(G)**，and plate cloning **(H** and **I)** validated the effect of SLC7A11 restoration on proliferative capacity in CRC cells interfering with LPCAT2. Scale bar: 100 μm.

* P < 0.05, ** P < 0.01, *** P < 0.001, **** P < 0.0001.

**Supplementary Figure 4. LPCAT2 is responsible for regulating the expression of SLC7A11.**

**(A)** Intersection of the top 240 differentially downregulated genes in HCT116 and DLD1 with high expression of LPCAT2. **(B)** GO enrichment analysis of co- downregulated genes and 28 genes involved in metabolic regulation. **(C)** 25 intersecting genes in Supplementary Figure 4A and 28 differentially expressed genes involved in metabolic process. **(D and E)** Grayscale analysis of protein bands using imageJ.

* P < 0.05, ** P < 0.01, *** P < 0.001.

**Supplementary Figure 5. LPCAT2 induces CRC cells ferroptosis.**

**(A)** MDA levels upon LPCAT2 overexpression and knockdown in basal conditions or in combination with any ferroptosis inducer. **(B)** IHC staining with apoptosis maker BCL2 and cleaved Caspase 3 in subcutaneous tumor tissue of nude mice. **(C)** LPCAT2 protein expression in PRMT1 knockdown cells.

* P < 0.05, ** P < 0.01, *** P < 0.001, **** P < 0.0001, ns indicates no statistical significance.

**Supplementary Figure 1. scRNA-seq identifies LPCAT2-positive tumor cell subsets.**

**
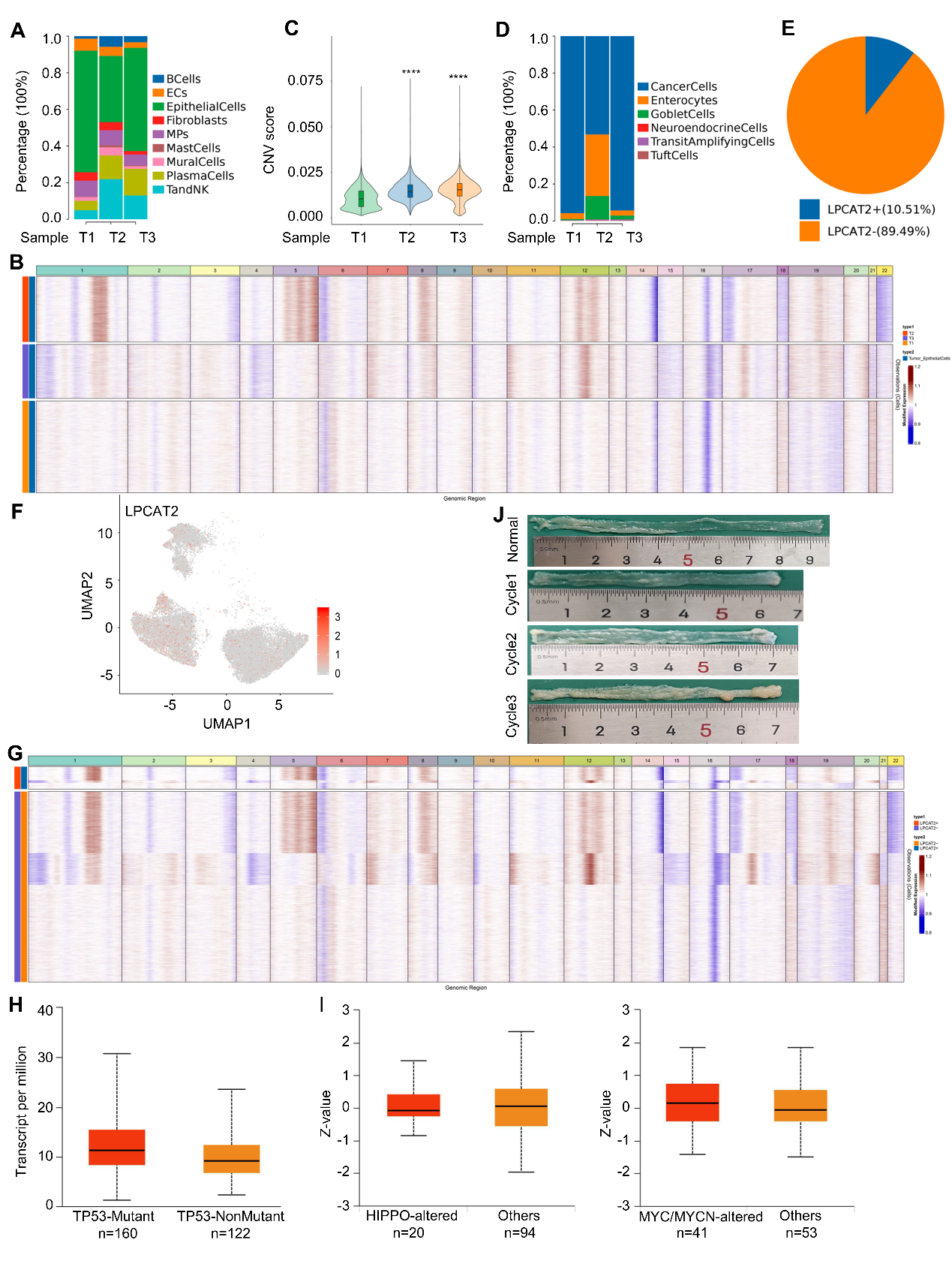
**

**Supplementary Figure 2. LPCAT2 influences the proliferation of CRC cells.**

**
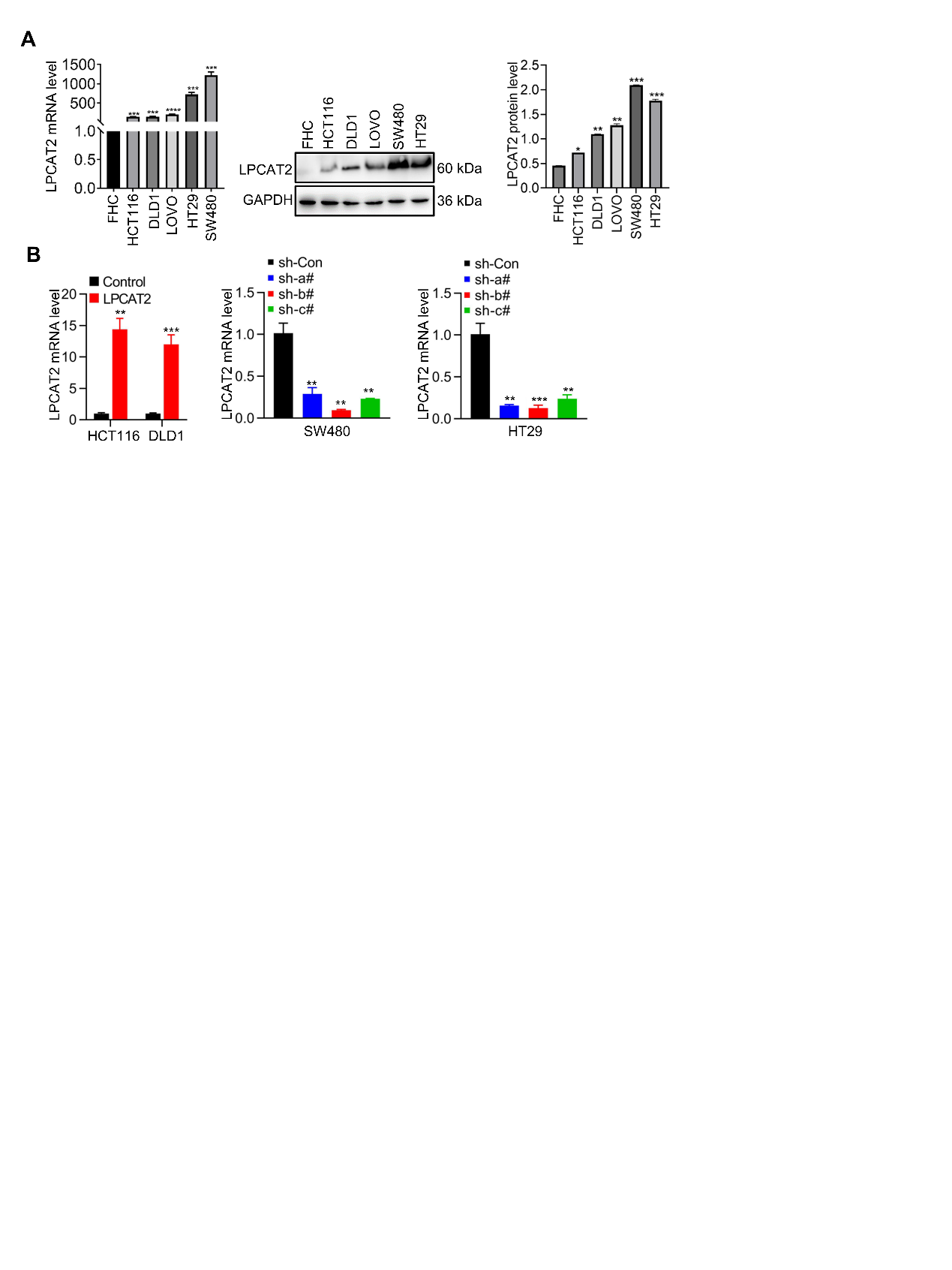
**

**Supplementary Figure 3. LPCAT2 induces ferroptosis in CRC cells through regulating SLC7A11.**

**
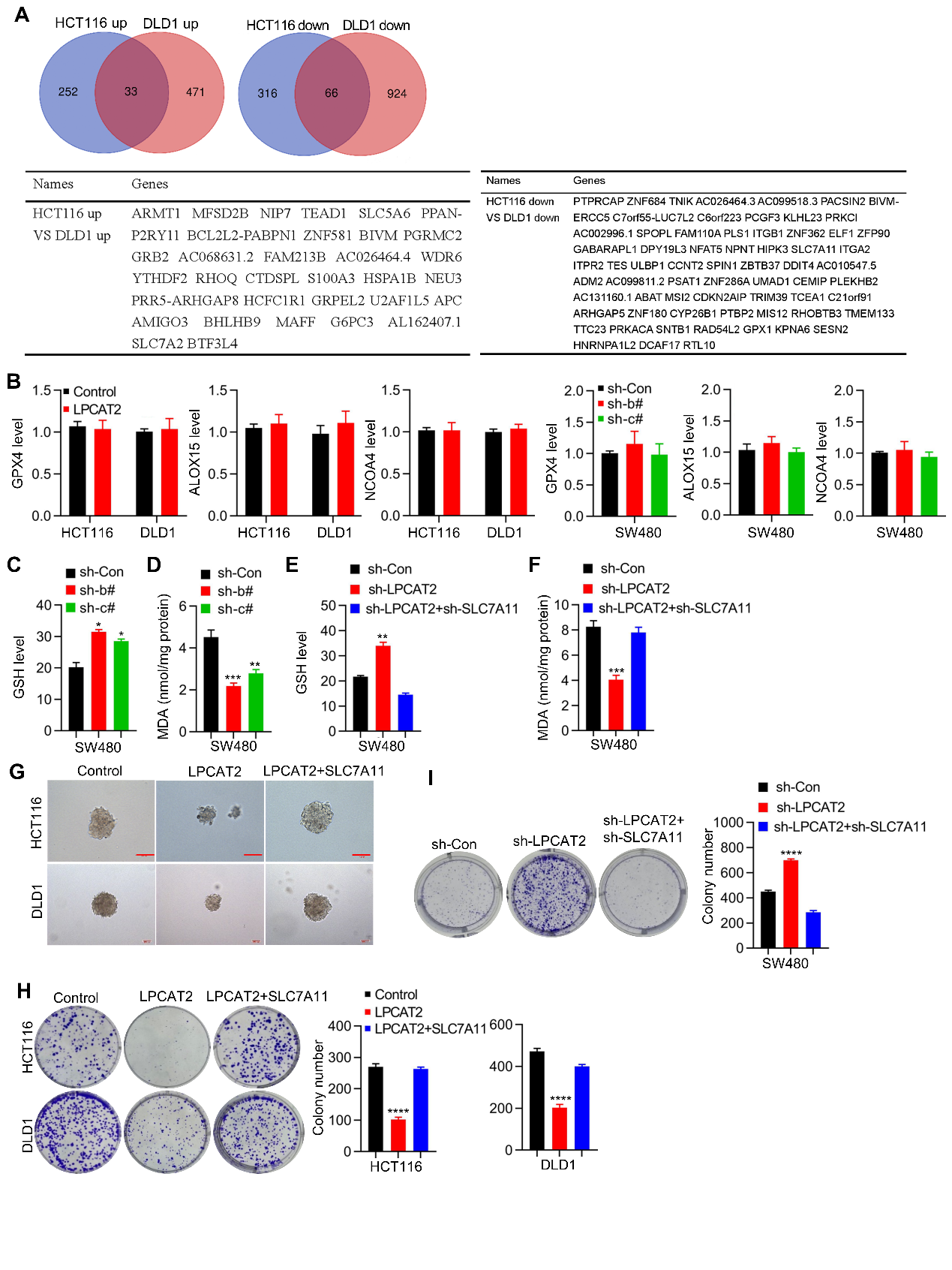
**

**Supplementary Figure 4. LPCAT2 is responsible for regulating the expression of SLC7A11.**

**
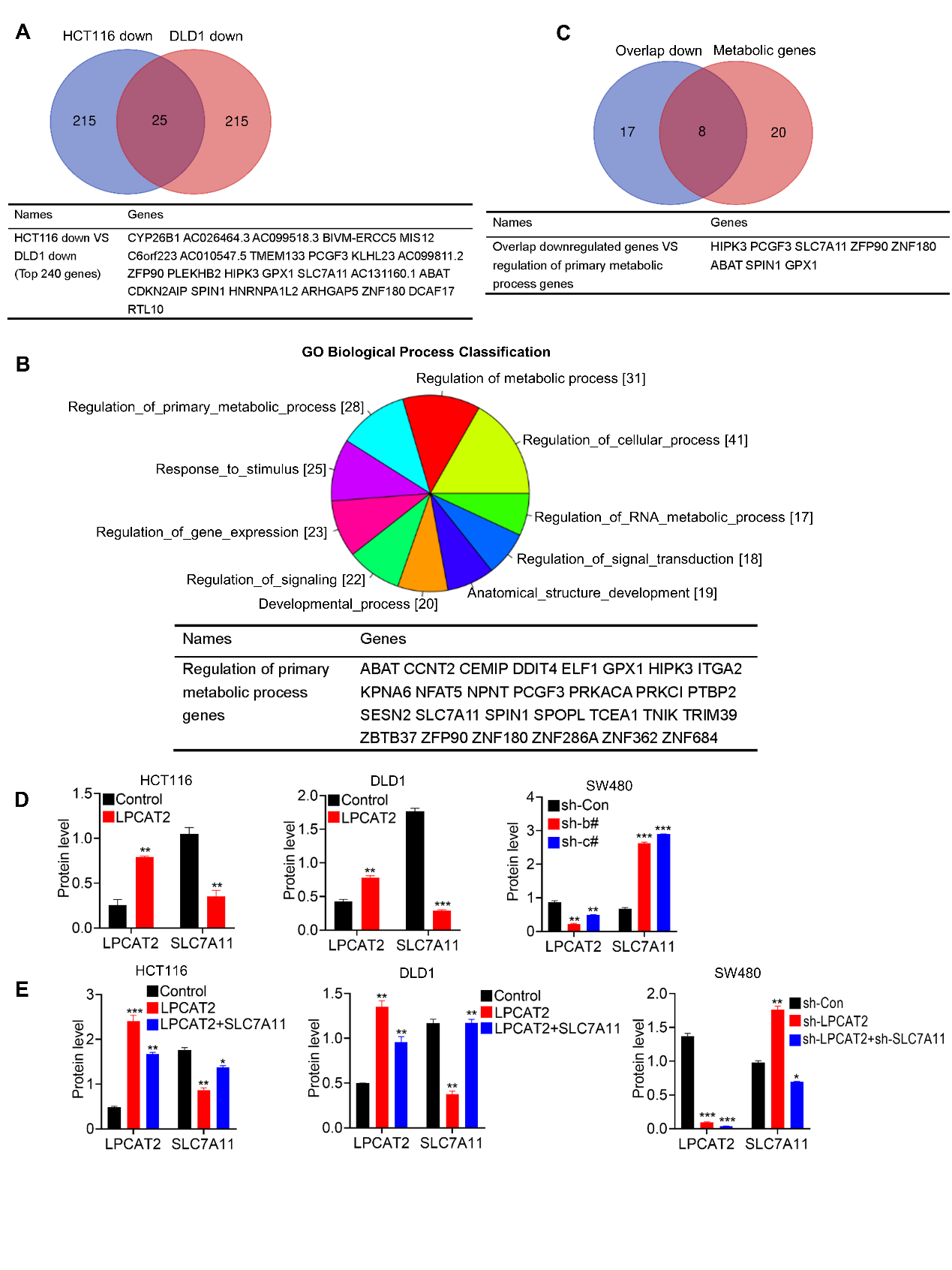
**

**Supplementary Figure 5. LPCAT2 induces CRC cells ferroptosis.**


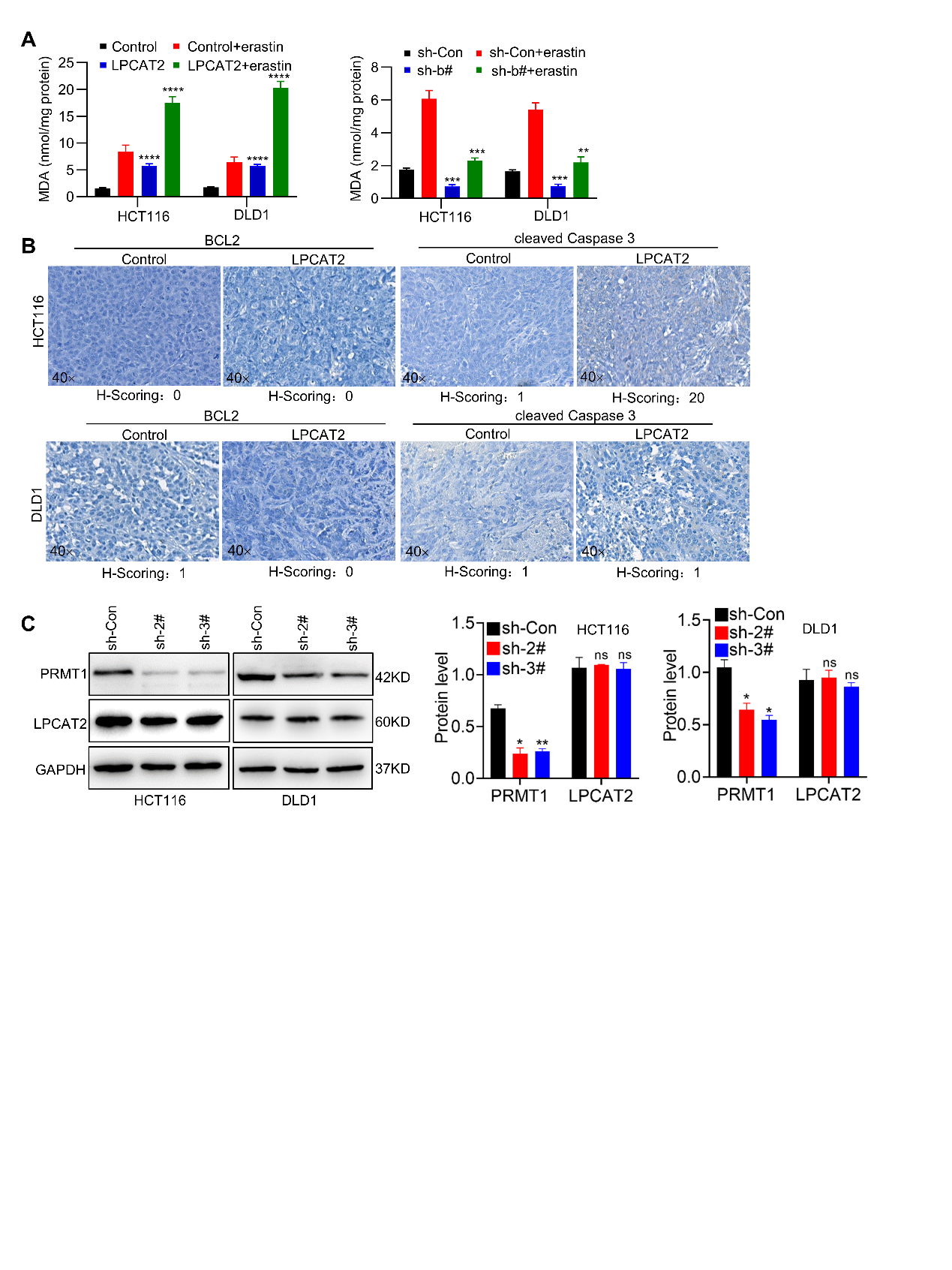

Supplement: Supplementary file 1 — supplementary materials [file 41388_2024_2996_MOESM1_ESM.docx]
